# Diagnosis and Management of Esophagogastric Varices

**DOI:** 10.3390/diagnostics13061031

**Published:** 2023-03-08

**Authors:** Socrate Pallio, Giuseppinella Melita, Endrit Shahini, Alessandro Vitello, Emanuele Sinagra, Barbara Lattanzi, Antonio Facciorusso, Daryl Ramai, Marcello Maida

**Affiliations:** 1 Department of Clinical and Experimental Medicine, University of Messina, 98100 Messina, Italy; socratep@tin.it; 2 Human Pathology of Adult and Child Department, University of Messina, 98100 Messina, Italy; 3 Gastroenterology Unit, National Institute of Gastroenterology “S. de Bellis” Research Hospital, Castellana Grotte, 70013 Bari, Italy; 4 Gastroenterology and Endoscopy Unit, S. Elia-Raimondi Hospital, 93100 Caltanissetta, Italy; 5 Gastroenterology and Endoscopy Unit, Fondazione Instituto San Raffaele Giglio, 90015 Cefalù, Italy; 6 Gastroenterology and Emergency Endoscopy Unit, Sandro Pertini Hospital, 00100 Rome, Italy; 7 Gastroenterology Unit, Department of Medical Sciences, University of Foggia, 00161 Foggia, Italy; 8 Gastroenterology & Hepatology, University of Utah Health, Salt Lake City, UT 84132, USA

**Keywords:** esophageal varices, gastric varices, portal hypertension, gastrointestinal bleeding, endoscopy

## Abstract

Acute variceal bleeding (AVB) is a potentially fatal complication of clinically significant portal hypertension and is one of the most common causes of acute upper gastrointestinal bleeding. Thus, esophagogastric varices represent a major economic and population health issue. Patients with advanced chronic liver disease typically undergo an upper endoscopy to screen for esophagogastric varices. However, upper endoscopy is not recommended for patients with liver stiffness < 20 KPa and platelet count > 150 × 10^9^/L as there is a low probability of high-risk varices. Patients with high-risk varices should receive primary prophylaxis with either nonselective beta-blockers or endoscopic band ligation. In cases of AVB, patients should receive upper endoscopy within 12 h after resuscitation and hemodynamic stability, whereas endoscopy should be performed as soon as possible if patients are unstable. In cases of suspected variceal bleeding, starting vasoactive therapy as soon as possible in combination with endoscopic treatment is recommended. On the other hand, in cases of uncontrolled bleeding, balloon tamponade or self-expandable metal stents can be used as a bridge to more definitive therapy such as transjugular intrahepatic portosystemic shunt. This article aims to offer a comprehensive review of recommendations from international guidelines as well as recent updates on the management of esophagogastric varices.

## 1. Introduction

Esophageal varices (EV) are one of the most common causes of acute upper gastrointestinal bleeding (UGIB) with varying prevalence worldwide [1,2]. They are the leading cause of death from UGIB. Acute variceal bleeding (AVB) is a potentially fatal complication of clinically significant portal hypertension (CSPH) and represents an important economic and population health issue.

EV are the seventh most common cause of GI bleeding in the United States, according to the literature [3]. The prevalence of schistosomiasis has been linked to EV in certain parts of the developing world [4]. Cirrhosis is the most common cause of EV in the Western world, with up to 85% of cirrhotic patients developing EV at some point in their lives [5,6], the incidence varying with disease severity. In compensated cirrhosis, EV develop at an annual rate of 8% [7], with higher rates in decompensated cirrhosis.

The distal third of the esophagus is the most commonly affected by esophageal varices, but proximal varices can occur in conditions affecting extra-portal venous circuits [8,9,10]. One-third of EV patients develop AVB, with overall mortality from a first episode ranging from 10% in compensated cirrhosis to 70% in decompensated disease [6].

## 2. Pathophysiology

Portal hypertension (PH) develops as a consequence of increased resistance to portal flow and is enhanced by the presence of increased portal collateral blood flow. 

The distinct site of obstruction or increased resistance can be sinusoidal (as in advanced chronic liver disease, “ACLD”), pre-sinusoidal (as in schistosomiasis, portal vein thrombosis), or post-sinusoidal (as in Budd–Chiari syndrome) (Table 1).

The increased resistance is mainly due to a combination of structural changes (distortion of the liver microcirculation by fibrosis, nodules, angiogenesis, and vascular occlusion) and dynamic changes (increased release of vasoconstrictors as angiotensin-II, norepinephrine, thromboxane A2 and endothelins, and the reduced production of vasodilators as nitric oxide).

Esophageal varices develop as a result of PH, which is traditionally assessed indirectly by determining the hepatic venous pressure gradient (HVPG): PH is defined as an HVPG > 5 mmHg, while CSPH is defined in presence of a gradient > 10 mmHg [7,9,10].

This is accomplished by measuring the pressure in the hepatic vein (HV) in two different settings. A balloon catheter is inserted into the jugular or femoral vein and advanced to the heart valve. The pressure of the HV is measured while the balloon is deflated and the catheter floats freely inside the vein. This determines the free HV pressure (FHVP). The balloon is then inflated until the HV is completely occluded. This creates a fluid column behind the balloon, which determines the wedged HVP (WHVP). The HVPG represents the gradient between the portal vein and intra-abdominal vena cava pressure and is the difference between the WHVP and FHVP. The HVPG has the advantage of not being affected by changes in intra-abdominal pressure [11].

## 3. Diagnosis and Risk Stratification

A physical examination may reveal signs of PH (e.g., caput medusa, enlarged hemorrhoids, platypnea, orthodeoxia, or hepatosplenomegaly). A Doppler ultrasound can show collateral circulation or portal flow reversal. Splenorenal shunts, dilated left and short gastric veins, and umbilical vein recanalization may also be seen in computed tomography (CT) and magnetic resonance imaging (MRI).

Despite the presence of clinical and/or imaging findings of PH, the gold standard for the diagnosis of EV and gastric varices (GV) is esophagogastroduodenoscopy (EGD).

The primary goal of EGD is the diagnosis and risk stratification of EV and GV by determining the size and high-risk stigmata.

**Figure 1:**
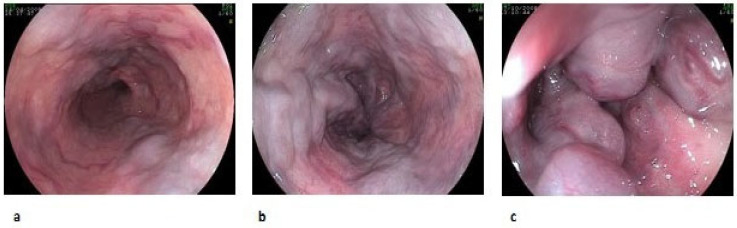
Esophageal varices according to size: F1 (**a**), F2 (**b**), F3 (**c**).

**Figure 2:**
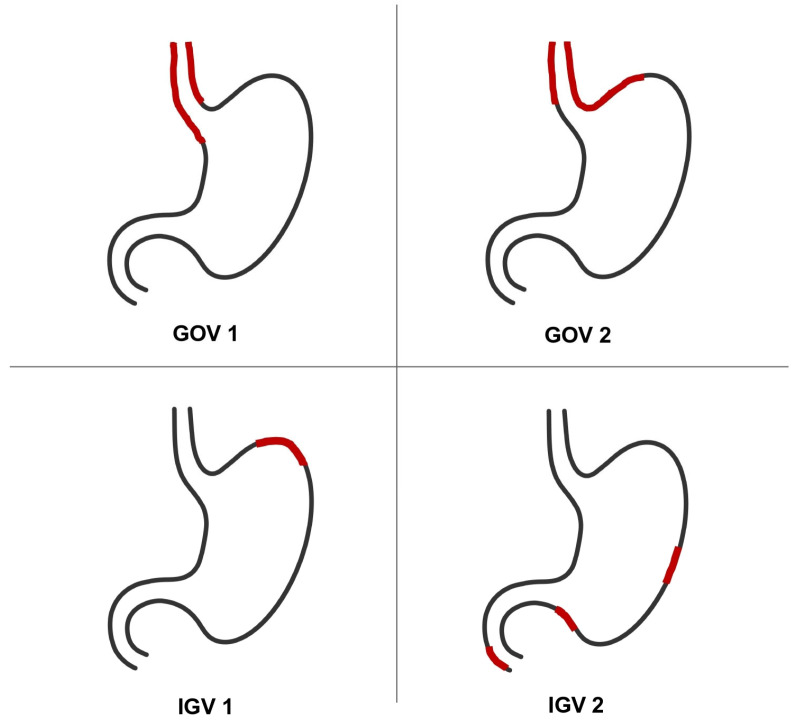
Illustration of different types of gastric varices according to the Sarin classification (GOV: gastroesophageal varices; IGV: isolated gastric varices) [13].

Esophageal varices are classified by size (small, medium, or large) and by the presence of red wale marks (Figure 1, Table 2) [12], while GV are classified as gastroesophageal varices (GOV) or isolated gastric varices (IGV) (Figure 2, Table 3) [13].

Elastography has been introduced in recent decades as a non-invasive method of determining the degree of liver stiffness. According to some studies, liver stiffness combined with platelet count accurately identifies patients with a low (5%) risk of EV in patients with compensated cirrhosis [14,15].

As a consequence, Baveno VII guidelines do not recommend upper endoscopy for the screening of EV in patients with liver stiffness less than 20 kPa and platelet counts greater than 150 × 10^9^/L [10].

This algorithm can be used to rule out varices that need to be treated with primary prophylaxis [16].

Notably, for patients with virally induced liver disease (i.e., HCV, HBV, etc.), the Baveno VI criteria (i.e., liver stiffness measured (LSM) < 20 kPa and PLT > 150 × 10^9^/L) can be used to manage ACLD after the primary etiological factor has been removed, thereby ruling out high-risk varices in patients with compensated liver disease who achieved SVR and viral suppression [10].

A new statement added to Baveno VII recommends that patients with compensated ACLD on nonselective beta-blocker (NSBB) therapy who have no visible CSPH (LSM 25 kPa) after the removal/suppression of the primary etiological factor undergo a repeat EGD within 1–2 years [10].

The accuracy of EGD in the detection and characterization of EV can be further improved by integrating artificial intelligence (AI).

Chen and colleagues used a convolutional neural network (CNN) to assess the accuracy of the endoscopically assisted detection and risk stratification of EV [17]. The authors showed that AI was associated with higher accuracy for detecting esophageal and GV compared to endoscopists only (97% vs. 93.94%, *p* < 0.01; 92% vs. 84.43%, *p* < 0.05, respectively). AI also showed superiority in identifying red wale signs for both EV and GV compared to endoscopists only (84.21% vs. 73.45%, *p* < 0.01; 85.26% vs. 77.52%, *p* < 0.05, respectively) [17].

Similarly, machine learning (ML) can be useful in refining the prediction of EV.

An ML-based algorithm showed to be effective in the prediction of EV and those needing treatment in patients with cirrhosis, avoiding unnecessary EGDs [18].

In the same line, a recent study showed that, when compared with the Baveno VI criteria, a novel ML-based model was effective in sparing more EGDs (52.6% vs. 29.4% in the training cohort; 58.1% vs. 44.2% in the validation cohort; *p* < 0.001) in patients with compensated cirrhosis [19].

While endoscopy is regarded as an invasive method for evaluating varices, other alternative tests have been evaluated over time.

Video capsule endoscopy (VCE) has been proposed as an alternative method for grading EV (especially the esophageal capsule system). A meta-analysis of 17 studies discovered that the diagnostic accuracy for grading medium to large varices was 92%, implying that VCE may be useful in patients who would prefer an alternative to endoscopy or in cases where endoscopy is contraindicated [20]. Endoscopic ultrasound (EUS) is a significant advancement in the field of advanced endoscopy, having progressed from a diagnostic tool to a real-time therapeutic modality.

The luminal gastrointestinal (GI) tract offers a unique opportunity to access multiple vascular structures, particularly in the mediastinum and abdomen, allowing for the real-time visualization of various structures by differentiating tissue densities and vascularity while avoiding radiation exposure.

With the addition of Doppler and contrast-enhanced capabilities, EUS allows for the real-time visualization of blood flow in vessels throughout the GI tract. Endoscopic accessories and interventional devices such as fine-needle aspiration (FNA) and fine-needle biopsy (FNB) needles are used during EUS-guided interventions [21,22].

Similarly, CT scans have shown approximately 90% sensitivity in the identification of EV, which were later determined to be large varices on endoscopy; however, the specificity was only 50%. The agreement between radiologists was good regarding the size of the varices (Kappa = 0.56) and exceeded the agreement between endoscopists (Kappa = 0.36). Nevertheless, CT is non-invasive and significantly more cost-effective compared to endoscopy, irrespective of the size of the varices [23].

## 4. Prevention and Management of Variceal Bleeding

The endoscopic management of EV can be divided into three scenarios: the role in preventing first variceal bleeding (primary prophylaxis), the treatment of AVB, and prophylaxis for re-bleeding after the first hemorrhaging event (secondary prophylaxis).

### 4.1. Screening of Esophageal Varices

**Figure 3:**
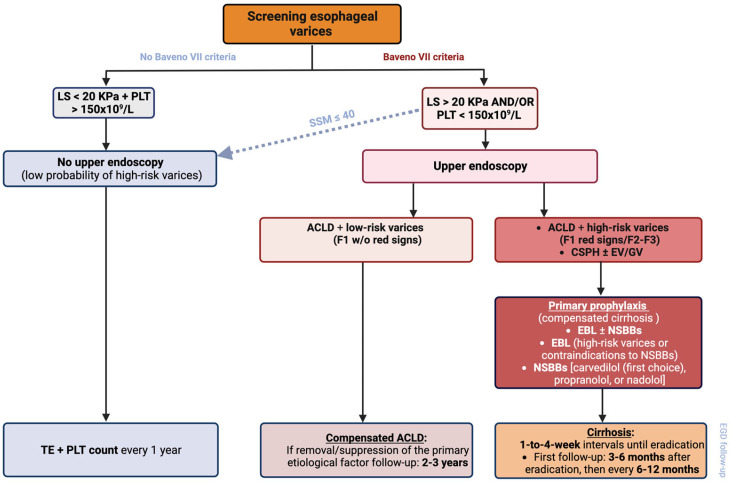
Screening and primary prophylaxis of esophageal varices.

Upper GI endoscopy should be used to identify high-risk EV (medium or large EV, or small EV with red wale marks) in patients with decompensated ACLD and LSM ≥ 20 KPa or platelet count ≤ 150 × 10^9^/L. Patients with compensated cirrhosis who are not candidates for NSBBs (e.g., contraindication/intolerance) should have a variceal screening endoscopy in cases of LSM ≥ 20 kPa or a platelet count ≤ 150 × 10^9^/L (Figure 3) [10].

Patients who are not candidates for screening endoscopy can be monitored with yearly TE and platelet counts. Furthermore, in patients who are not candidates for NSBBs (contraindication/intolerance) and would traditionally require endoscopy based on the Baveno VII criteria, spleen stiffness measurement (SSM) ≤ 40 kPa by TE can be used as a surrogate marker to identify those with a low probability of high-risk varices, thereby avoiding endoscopy [10].

On the other hand, the European Society of Gastrointestinal Endoscopy (ESGE) guidelines do not recommend the use of VCE for the screening of EV [20,24,25].

Because patients with porto-sinusoidal vascular disorder (PSVD) cannot use the non-invasive Baveno VII criteria for screening EV in cirrhotic patients, endoscopy is typically required at the time of PSVD diagnosis [10]. The frequency of endoscopic screening for EV should adhere to the same guidelines as those for liver cirrhosis.

### 4.2. Primary Prophylaxis of Esophageal Varices Bleeding

Primary prophylaxis is especially important in compensated patients with CSPH and/or EV or GV because they are at high risk of decompensating (Figure 3) [10]. Patients with ACLD and high-risk varices should receive primary prophylaxis. Both NSBB therapy and endoscopic band ligation (EBL) have been shown to significantly reduce the risk of the first episode of variceal bleeding. 

Treatment with NSBBs (propranolol, nadolol, or carvedilol) should be considered for the prevention of decompensation in patients with CSPH. In particular, carvedilol should be preferred as the first choice in compensated cirrhosis [10], since it is more effective at reducing HVPG and preventing decompensation, has better tolerance than traditional NSBBs, and has been demonstrated to improve survival [26].

NSBBs or carvedilol should, however, be reduced or discontinued in patients with ascites who have persistently low blood pressure (systolic blood pressure 90 mmHg or mean arterial pressure 65 mmHg) and/or HRS-AKI. NSBBs can be restarted once blood pressure returns to normal and/or HRS-AKI resolves. A screening endoscopy is not required in patients with compensated cirrhosis who are taking NSBBs for primary prophylaxis.

If an endoscopy does not reveal high-risk varices and the patient is unable to receive NSBB therapy, ESGE recommends a surveillance endoscopy every 2 years if the underlying liver disease is active or every 3 years if the underlying liver disease is quiescent (Table 4) [25].

Baveno VII guidelines recently recommended endoscopic band ligation to prevent first variceal bleeding in compensated patients with high-risk varices who have contraindications or intolerance to NSBBs [10].

In addition, while one study found that cyanoacrylate (CYA) injection was more effective than propranolol in preventing first bleeding in patients with large type 2 gastric varices or isolated type 1 gastric varices, no differences in survival were found [10].

There is currently no indication for compensated patients to undergo balloon-occluded retrograde (antegrade) transvenous obliteration (BRTO or BATO) or transjugular intrahepatic portosystemic shunt (TIPS) for primary prophylaxis of gastric variceal bleeding.

### 4.3. Management of Acute Esophageal Variceal Bleeding

Esophageal varices generally have an asymptomatic course until they leak or burst.

The rupture of esophageal varices presents with severe hemorrhage, characterized by hematemesis and/or melena, severe anemia, and possible loss of consciousness. This state represents a medical emergency and requires prompt intervention in an emergency unit.

#### 4.3.1. Hemodynamic Resuscitation

Hemodynamic resuscitation aims to improve tissue perfusion, correct intravascular hypovolemia, and prevent multiorgan failure [27]. However, there is still debate about the optimal rate of fluid resuscitation (aggressive vs. restrictive), particularly for esophagogastric variceal bleeding, with some evidence suggesting that a restrictive fluid resuscitation strategy may result in fewer AEs and may reduce mortality [28,29]. The optimal intravenous fluid for initial resuscitation is unknown, with crystalloids or colloids commonly used while blood product transfusion is assessed [30,31].

In both a large randomized controlled trial (RCT) and a meta-analysis of critically ill patients, using a “balanced” crystalloid solution (e.g., lactated Ringer’s solution) was shown to reduce both mortality and major adverse renal events when compared to saline [32]. 

Anesthetic practices during endoscopic procedures to treat esophageal varices are classified as non-anesthesiology sedation (NAS), which means that the level of patient sedation is entirely up to the endoscopist, who is usually assisted by a well-trained nurse, and those that require anesthesiology support, which is classified as Monitored Anesthesia Care (MAC) and Non-Operating Room Anesthesia (NORA). The aim of sedation for GI endoscopy is to relieve the patient’s anxiety and discomfort while ensuring a technically successful procedure [33].

Fresh frozen plasma transfusion is not recommended during an AVB episode, according to Baveno VII, because it will not correct coagulopathy and may lead to volume overload and the worsening of portal hypertension [10]. There is no evidence that platelet count or fibrinogen levels are associated with the risk of AVB failure to control bleeding or re-bleeding. If the bleeding cannot be stopped, the decision to correct the hemostatic abnormalities should be made on an individual basis. Recombinant factor VIIa and tranexamic acid are also not recommended in AVB [10].

Even if transfusion policy must consider other factors such as cardiovascular status, age, and ongoing bleeding, red blood cell transfusions should be performed conservatively, with a target hemoglobin level of 7–8 g/dL [10].

Anticoagulants should be temporarily discontinued in AVB patients until the hemorrhage is under control. The duration of discontinuation should be tailored to the strength of the indication for anticoagulation.

#### 4.3.2. Risk Stratification

According to the European Society of Gastrointestinal Endoscopy, patients with ACLD who present with suspected AVB should be risk stratified using the Child–Pugh and MELD scores, as well as the documentation of active/inactive bleeding at the time of upper GI endoscopy [25].

According to a meta-analysis of individual patient data, patients with a Child–Pugh score of B > 7 and C ≤ 13 points and active variceal bleeding at GI endoscopy (defined as variceal jet/oozing despite the use of vasoactive drugs) are at risk of a poor outcome and may benefit from preemptive TIPS placement [34].

Although there are concerns about the prognostic capacity of these variables due to subjectivity in evaluating the severity of ascites or hepatic encephalopathy, as well as the true risk of Child–Pugh B patients, some studies have shown that they are effective in classifying patient risk [35,36]. MELD ≥ 19 has also been used in several studies [37,38] to identify high-risk ACLD patients.

In addition, the European Society for the Study of the Liver (EASL) adopted the Chronic Liver Failure–Sequential Organ Failure Assessment (CLIF-SOFA), which is used to differentiate acute decompensation from acute-on-chronic liver failure (ACLF) [39]

A recent study showed that comparing MELD-Na, CPT, and CLIF-SOFA in predicting mortality in patients with variceal bleeding, CLIF-SOFA performed better than other scores, with an area under the receiver operating characteristic curve (AUROC) of 0.79 for 30- and 90-day mortality in patients with ACLF, while CPT performs better in patients with acute decompensation with an AUROC of 0.71 (0.58–0.82) for 30-day and an AUROC of 0.74 (0.61–0.85) for 90-day mortality [40].

Another study confirmed the CLIF-SOFA score as a significant prognostic factor of 28-day mortality in patients with ACLF and variceal bleeding (HR, 1.32; 95% CI, 1.19–1.46, *p* < 0.001) [41].

In the same line, the CLIF-SOFA score was also superior to MELD and CTP in predicting the in-hospital and 6-week mortality of patients with variceal hemorrhage after EBL.

In detail, the AUROCs of the CLIF-SOFA score, MELD score, and CTP for predicting in-hospital death were 0.964, 0.876, and 0.846. For predicting 6-week death, the AUROC values of the CLIF-SOFA score, MELD score, and CTP class were 0.943, 0.817, and 0.834 [42].

#### 4.3.3. Medical Therapy

1. Vasoactive agents

**Figure 4:**
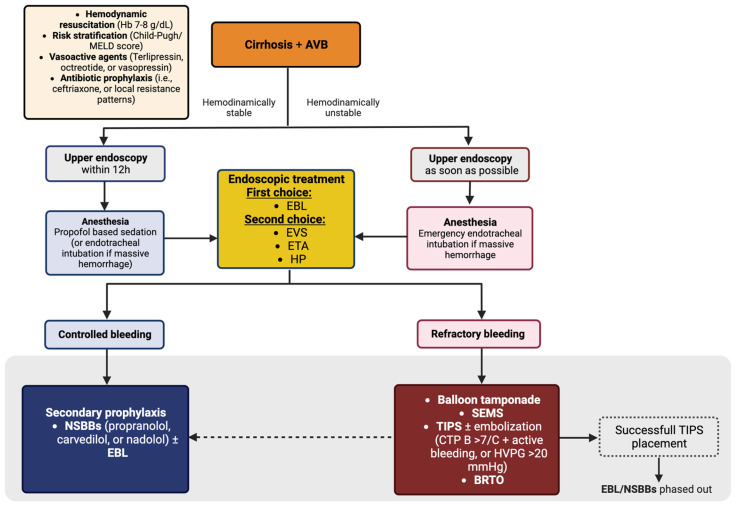
Management of acute esophageal variceal bleeding.

Baveno VII recommends that in cases of suspected variceal bleeding, vasoactive drugs (such as terlipressin and octreotide) be started as soon as possible and continued for 2–5 days (Figure 4) [10].

Several systematic reviews and meta-analyses [43,44] evaluated the efficacy and safety of vasoactive agents in AVB, concluding that vasoactive agents outperform no vasoactive treatment in terms of in-hospital mortality, overall mortality, variceal bleeding control, variceal re-bleeding, and blood transfusion requirement. Octreotide appears to be as effective as terlipressin and vasopressin, but with fewer side effects. Vasopressin is no longer used because of its extra-splanchnic vasoconstrictive properties and high AE profile. Vasoactive agents have also been shown to significantly lower the rate of early re-bleeding after successful endoscopic hemostasis (within 5 days of AVB) [45].

2. Antibiotic prophylaxis

Bacterial infections are common in compensated CSPH patients and can cause decompensation. Patients with ACLD who present with AVB are predisposed to bacterial infection, particularly respiratory tract infection [46]. Bacterial infection increases the risk of re-bleeding and the overall mortality rate. In a multicenter retrospective cohort study, 371 patients with cirrhosis and AVB received antibiotic prophylaxis. Despite antibiotic prophylaxis, 14% of patients developed a bacterial infection within 14 days (of which more than half of all infections were caused by respiratory infections) [46].

Furthermore, some studies show that in patients treated under standard conditions, the likelihood of bacterial infections is significantly lower compared to patients treated under emergency conditions [47].

Antibiotic prophylaxis is an essential part of treatment for patients with ACLD who present with UGIB, according to Baveno VII guidelines, and should be started right away. In patients with advanced cirrhosis in hospital settings with a high prevalence of quinolone-resistant bacterial infections and in patients who have previously received quinolone prophylaxis, intravenous ceftriaxone 1 g/24 h for up to seven days should be considered. These recommendations, however, should always be consistent with local resistance patterns and antimicrobial policies [10].

#### 4.3.4. Timing of Endoscopy

Due to differing definitions of “early” and “late” endoscopy and study conclusions, the optimal timing of upper GI endoscopy in patients with AVB is debatable, implying a lack of high-level evidence for guideline recommendations.

Upper endoscopy should be performed on patients with suspected AVB within 12 h of presentation after hemodynamic resuscitation. If the patient is unstable, an endoscopy should be performed as soon as possible (Figure 4) [10]. Overall mortality was significantly lower in the early endoscopy (12 h) group compared to the delayed endoscopy (>12 h) group in a systematic review/meta-analysis of 2824 patients with ACLD and AVB by Bai et al. [48].

There is no evidence that has been identified that has evaluated the INR value at the time of patient presentation and its influence on the timing of upper GI endoscopy in the setting of AVB. According to the ESGE guidelines, the timing of upper GI endoscopy in patients with suspected AVB should not be influenced by INR levels at the time of presentation [25,49].

#### 4.3.5. Anesthesia

1. Elective procedures

Following a pre-procedure evaluation of the patient using the ASA classification as well as the identification of all risk factors, the most appropriate type of anesthesia for each patient should be chosen. Patients undergoing endoscopic procedures for the diagnosis and treatment of EV are typically those with liver cirrhosis with an increased risk of sedation-related complications and the need for anesthesia-directed sedation assistance [47,50,51].

Propofol is especially appealing in patients with liver disease because it has a short duration of action, is quickly metabolized, and has a better profile than benzodiazepines such as midazolam [52,53].

Midazolam is commonly used during the NAS routine, but studies have shown that combining low-dose midazolam and propofol results in a better sedative effect and endoscopist satisfaction than midazolam alone [47].

Furthermore, the addition of opioids to sedatives could be considered to improve analgesia and reduce visceral pain. Oxycodone and midazolam or oxycodone and propofol have a sedative and analgesic effect, which inhibits the stress response. Fentanyl, on the other hand, may cause respiratory depression, choking, and stiffness of the chest wall muscles [54,55].

2. Emergency procedures

There is no universally accepted approach to the level of monitoring and anesthetic support required for patients undergoing acute EGD for UGIB, as some are performed under general anesthesia with endotracheal intubation, while others are performed under MAC [56].

Patients with variceal bleeding typically have more comorbidities and a higher mortality rate than patients without variceal bleeding and, as a result, many endoscopists are hesitant to perform sedation in these patients [57]. However, recent research has shown that propofol-based sedation can be used to keep patients stable during the procedure and ensure a successful outcome with a low-risk profile [58].

It is stated that sedation may be ineffective if the patient’s general condition deteriorates or if there is hemodynamic instability. Although aspiration is the primary concern with emergency procedures for UGIB, sedation endoscopy did not increase the incidence of this type of adverse event when compared to non-sedation endoscopy [59]. Preventing aspiration with prophylactic endotracheal intubation for airway protection is not effective, resulting in no significant differences in mortality and the length of hospitalization and a higher rate of adverse events, particularly in patients with pre-existing cardiac disease, which is usually attributed to the medications used for sedation and analgesia [60,61,62].

Excessive sedation, altered consciousness, desaturation, airway obstruction, or aspiration during the procedure (i.e., during active bleeding), on the other hand, may necessitate emergency endotracheal intubation [10,63]. Endotracheal intubation should be provided in cases of massive hemorrhage, and it is typically performed on the patient in the supine position, followed by a shift of decubitus to the left lateral position. Since it is dangerous to move patients after anesthesia induction, and with recent advances in video technology, intubation could be performed directly with the patient in the left lateral position using the video-laryngoscope [64].

Extubation should be done as soon as possible after endoscopy [10].

#### 4.3.6. Endoscopic Treatment

Endoscopy has a key role in the management of EV bleeding (Figure 4).

Notably, an on-call GI endoscopist proficient in endoscopic hemostasis and on-call support staff with technical expertise in the use of endoscopic devices are recommended [10]. Moreover, the medical team may find it beneficial to have the interventional radiology (IR) staff alerted early in cases of uncontrolled bleeding necessitating TIPS.

1. Endoscopic variceal ligation

EBL is the preferred type of endoscopic therapy for AVB [65,66,67,68,69]. The current EBV method makes use of a multiband device [65,66]. An endoscope cap-assisted ligation device deploys an elastic band around the varix after it has been suctioned into the cap by turning a firing device attached to the external biopsy valve port. This causes varix strangulation and hemostasis, which is followed by intravascular thrombus formation, necrosis, fibrosis, and varix obliteration. Bands are first placed distally, focusing on varices with recent bleeding, platelet plugs, or active bleeding stigmata. Bands are placed helically from the distal to the proximal esophagus. Due to impaired vision, this may be difficult in the presence of active bleeding.

EBL is effective in controlling active variceal bleeding in approximately 90% of cases [66]. Many randomized controlled trials (RCTs) have compared the efficacy of EBL to esophageal variceal sclerotherapy (EVS) for AVB, and a meta-analysis found that EBL had lower re-bleeding and mortality rates than EVS [70,71]. Another meta-analysis discovered that EBL was superior to EVS in terms of re-bleeding, complications, and variceal eradication, but there was no significant difference in mortality [72]. EBL should be repeated at regular intervals after AVB treatment until the varices are completely eradicated.

In 2% to 20% of patients, EBL complications include transient dysphagia, retrosternal pain, post-banding bleeding, esophageal stricture, esophageal ulcerations, esophageal perforation, and infection [47,66]. A recurring bleeding vessel or post-banding ulceration can cause re-bleeding. Post-banding ulcer bleeding affects 3.6% to 15% of patients [73].

2. Esophageal Variceal Sclerotherapy

In contrast to EBL, which is mechanical, the mechanism of EVS is chemical [74].

The injection of a sclerosant agent (e.g., sodium tetradecyl sulfate, ethanolamine oleate, sodium morrhuate, polidocanol, or absolute alcohol) is performed immediately adjacent to or within the varix, causing inflammation and thrombosis [66].

EVS was the first endoscopic treatment to be shown to be superior to balloon tamponade (BT) or vasoactive drugs in the past [75]. Although EVS was used for endoscopic therapy in the 1980s, it was largely replaced by EBL in the 1990s after studies revealed fewer re-bleeding episodes and adverse events [76].

In up to 40% of patients, sclerosing causes fever, dysphagia, retrosternal discomfort, injection-induced bleeding, esophageal ulceration with bleeding, pleural effusion, pneumothorax, mediastinitis, and infection (including spontaneous bacterial peritonitis) [77]. Recurrent variceal bleeds or post-injection ulceration can cause re-bleeding. When compared to EBL, EVS has been linked to a higher risk of complications, including pleuropulmonary complications, bleeding, and infection [78].

3. Endoscopic tissue adhesives

Endoscopic injection tissue adhesives (ETA) are another method of treating varices [79,80]. Initially, this technique was used to treat gastric varices and ectopic varices rather than EV. CYA tissue glue causes endothelial injury and venous obturation, which leads to hemostasis [81]. The bleeding was successfully controlled in 75% of patients with Child–Pugh class C cirrhosis and AVB treated with CYA. A prospective study of cirrhotic and AVB patients who were not amenable to EBL due to severe bleeding and were randomly assigned to EVS or ETA discovered that bleeding arrest was significantly higher with ETA, with no significant differences in the order of re-bleeding. EVS and ETA should be considered when EBL is technically difficult [7,10,82].

4. Hemostatic powders

Hemostatic powders (HP) have recently been introduced for the treatment of gastrointestinal bleeding, with overall good efficacy and safety [83]. They can be sprayed using a specialized catheter. Hemospray^®^ is an inert mineral-based compound that absorbs water when it comes into contact with blood and becomes adherent to the bleeding site. However, HP has primarily been studied in the context of ulcer and tumor bleeding. A recent study found that 13 (7%) patients treated with Hemospray^®^ or Endoclot^®^ for GI bleeding had varices [84]. A short-term success rate of 85% and a long-term success rate of 56% were demonstrated. In a trial of cirrhotic patients with AVB, comparing the early (2 h) application of HP with early elective endoscopy (12–24 h), the authors demonstrated a significant improvement in hemostasis [10].

Despite this, hemostatic powder (HP) and endoscopic tissue adhesives cannot be recommended as first-line endoscopic therapy due to a lack of evidence [10].

#### 4.3.7. Refractory Bleeding

Up to 20% of AVB episodes can be refractory to standard therapy and are associated with high mortality. The causes are as follows: (1) massive bleeding that precludes visualization or endoscopic therapy, (2) an inability to stop the bleeding, and (3) prompt re-bleeding.

A high mortality rate of 30% to 50% is associated with cases of refractory bleeding [85]. Bridge therapy includes balloon tamponade (BT), esophageal stent placement, or TIPS.

1. Balloon tamponade

Balloon tamponade was developed to control AVB and has been used to provide temporary hemostatic control until more definitive therapy can be administered [67].

It achieves hemostasis in up to 80% of patients, but it is associated with a high rate of serious adverse events and a mortality rate of around 20% [7]. Furthermore, BT should not last more than 24 h. In a retrospective study of 34 patients treated with BT after AVB, 59% survived until discharge and 95% received concurrent TIPS [86].

2. Esophageal stents

Self-expandable metal stents (SEMS), such as BT, can be used as a bridge to EBL or TIPS in refractory variceal bleeding [10,65].

Stents achieve hemostasis by directly compressing the varices. When compared to BT, they have comparable efficacy but may be more expensive.

A systematic review and meta-analysis of five studies using specialized SEMS found that stents had a 93.9% hemostasis rate and a 13.2% re-bleeding rate (after stent placement) [87].

SEMS controlled bleeding in 79% of patients in a multicentric retrospective study of refractory AVB [86]. The study also found that 38.2% of people died with the stent in place, and 47% died from bleeding.

Stent migration is the most common complication, but esophageal ulceration has also been reported [88].

3. TIPS

In high-risk patients who meet any of the following criteria, a transjugular intrahepatic portosystemic shunt with polytetrafluoroethylene (PTFE)-covered stents is recommended as an early option: Child–Pugh class C or B greater than 7 with active bleeding at initial endoscopy (or HVPG greater than 20 mmHg at the time of AVB) [10]. Nonetheless, unless a liver transplant is planned in the near future, TIPS may be ineffective in patients with Child–Pugh > 14 cirrhosis, or a MELD score > 30 and lactate > 12 mmol/L [10]. TIPS should only be used in such patients on a case-by-case basis.

Moreover, TIPS is recommended as a salvage option in refractory bleeding when a combination of pharmacological and endoscopic therapy fails to control variceal bleeding.

Several retrospective studies have been conducted to assess the role of salvage TIPS, despite the fact that there are no high-level RCTs. In a review of 15 studies, Vangeli et al. reported on outcomes following the use of TIPS as salvage therapy [89]. Technical success was 100%, with up to 16% variceal re-bleeding and a 75% mortality rate [88]. TIPS failed in 16% of 144 patients with refractory EV bleeding in a retrospective study. At 6 weeks and 12 months, the mortality rates were 36% and 2%, respectively. All patients with a Child–Pugh score of 13 or higher died [90].

These findings confirmed that in patients who continue to bleed despite vasoactive and endoscopic therapy, urgent rescue intervention with TIPS should be considered early during their clinical course.

According to Baveno VII, lowering the absolute portal pressure gradient (PPG) to less than 12 mmHg is associated with near-complete protection from portal hypertensive bleeding in patients with variceal bleeding undergoing TIPS and is the preferred target for achieving hemodynamic success. A 50% decrease in PPG from the pre-TIPS baseline may also be advantageous.

Notably, TIPS may be combined with embolization to control bleeding or reduce the risk of recurrent variceal bleeding from gastric or ectopic varices, particularly when portal flow remains diverted to collaterals despite a decrease in portosystemic pressure gradient [10].

In patients with type 2 GOV, type 1 IGV, and ectopic varices, balloon-occluded retrograde transvenous obliteration could be considered as an alternative to endoscopic treatment or TIPS, provided it is feasible (type and diameter of shunt) and local expertise is available [10,13].

4. Surgery

Emergency surgery has a limited role in the treatment of AVB.

Nonetheless, it may be considered as a rescue option in case of the failure of all previous non-surgical lines of treatment (including TIPS). Moreover, it is also an option for refractory AVB occurring in medical centers that do not have access to radiological interventions.

The main surgical procedures include total portosystemic shunt, partial shunt, selective shunt, and portal-azygos disconnection surgery. The most commonly used techniques of portal-azygos disconnection are lower esophageal transection, gastric fundus and lower esophageal transection with an automatic stapler, and the transgastric ligation of variceal bleeding (Boerema and Crile ligation techniques) [91]. The selection of specific surgical procedures should be assessed according to several factors, such as the timing of surgery, operative indications, etiology, liver function, the hemodynamic status of the patient, and the surgeon’s experience. Surgical procedures are not recommended for patients with decompensated liver disease with a Child–Pugh score of C.

### 4.4. Management of Acute Gastric Variceal Bleeding

Although acute gastric variceal bleeding is less common than esophageal bleeding, it is more severe, with higher associated mortality and treatment failure [92].

General measures for the management of acute hemorrhage, including hemodynamic resuscitation, the timing for upper endoscopy, and anesthesia are similar to those previously described for EV bleeding.

**Figure 5:**
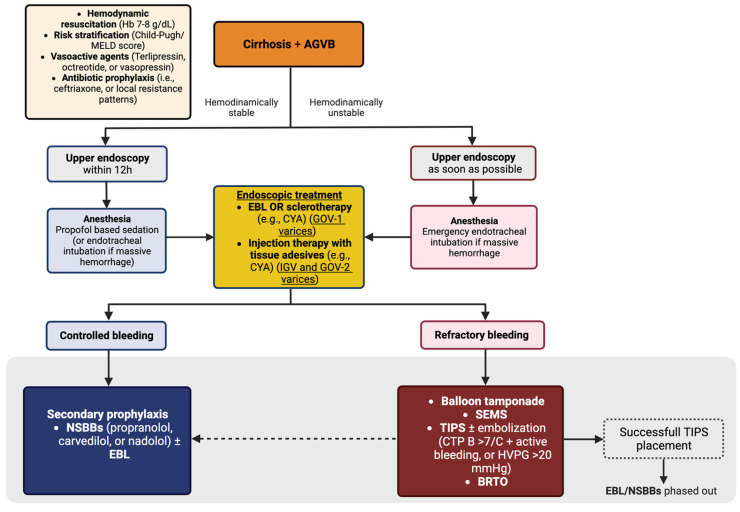
Management of acute gastric variceal bleeding.

Concerning endoscopic hemostasis, EBL or injection of tissue adhesives (e.g., CYA) are recommended for bleeding from GOV-1 varices, while injection therapy with tissue adhesives (e.g., CYA) is recommended for acute bleeding from IGV and GOV-2 varices (Figure 5) [10]. However, high-quality data on the best endoscopic treatment are scarce.

Qiao et al. reported on three RCTs that included 194 patients with active gastric variceal bleeding and compared endoscopic CYA injection to EBL [93]. Active bleeding was controlled at 79.5% in the EBL group and 93.3% in the CYA injection group. Re-bleeding was comparable between the two interventions for GOV, but CYA was superior for reducing re-bleeding.

A novel EUS-based method allows for the direct visualization and access of gastric varices for treatment and obliteration [94,95].

Romero-Castro et al. described the first EUS-guided CYA injection in 2007 [96]. Franco et al. studied 20 patients who had EUS-CYA for primary prophylaxis and found that obliteration was successful in all of them. Only one of them experienced recurrent bleeding [97]. Romero-Castro et al. reported on ten patients with active gastric variceal bleeding. They were successful with EUS-CYA in all cases [98]. Gubler described 40 patients who underwent EUS-CYA for either acute or prophylactic bleeding. Only three patients required TIPS or a liver transplant to be saved. All other cases were halted [99].

A single-center study compared the treatment of 40 patients with actively bleeding or high-risk GV with direct endoscopic injection of CYA to the treatment of 64 patients with EUS-guided injection of CYA. A greater number of variants were eliminated in the EUS-guided group. The volume of CYA injected was greater with direct endoscopic injection than with EUS-guided fine needle injection. After the procedure, the direct endoscopic injection group had a higher rate of GV re-bleeding [100].

In the last few years, EUS-guided coil injection, with or without CYA, was introduced. The coil can provide primary hemostasis while also retaining glue within the varix, lowering the risk of embolization [101]. Coil injection was first described by Romero-Castro et al. in 2010. There is a dearth of data on coil injection alone. Romero Castro et al. used an EUS coil for primary GV prophylaxis in four patients and discovered that coil placement eradicated varices in three of them without complications or migrations [102]. Bhat et al. published a large case series of 151 GV patients who received successful treatment with an EUS coil/CYA, with 125 having clinical or endoscopic/EUS follow-ups.

Of the 100 patients who had a follow-up EUS, 73 showed complete obliteration in a single procedure, 14 required additional treatments, 3 were unable to be obliterated, and 4 had residual varices detected at the time of follow-up [103]. This case study validates the efficacy of the EUS coil/CYA in the treatment of GV. Coil placement, with or without CYA, can have unfavorable outcomes. Romero-Castro et al. observed coil extrusion into the gastric lumen with mucosal scarring in 1 of 11 patients (9%).

Minor GI bleeds from the puncture site were reported in 50% of cases, and minor bleeding from coil or CYA extrusion was reported in 3% of cases [103].

Khoury et al. discovered that 10% of patients undergoing EUS coil had significant bleeding from the puncture site [104].

### 4.5. Secondary Prophylaxis and Follow-Up after First Bleeding Episode

To prevent recurrent variceal bleeding, patients recovering from a first episode of variceal bleeding should be treated with a combination of NSBBs and EBL. In patients who are not candidates for EBL, carvedilol, or traditional NSBBs, any of these therapies can be used alone, while TIPS should be considered in patients with recurrent ascites [10].

In patients who re-bleed despite traditional NSBBs or carvedilol and EVL, a transjugular intrahepatic portosystemic shunt is the treatment of choice [10].

The AASLD recommends 1-to-4-week intervals for EBL follow-up until eradication, with the first follow-up EGD performed 3 to 6 months after eradication and then every 6 to 12 months [7,66]. Endoscopic ultrasound probe results following EV eradication may be used to predict variceal recurrences [94].

Endoscopic ultrasound was used in a prospective cohort study to reveal the clinical potential for EV evaluation [105]. Because of the high risk of re-bleeding following initial AVB (60%), NSBB (propranolol or nadolol) combination therapy is preferred over EBL alone [7,10]. Given the severity of the re-bleeding and other PH complications, particularly hepatic encephalopathy, TIPS is the recommended rescue treatment if patients re-bleed despite combination therapy with EV and NSBB [106,107].

EBL and NSBB can be phased out after TIPS placement is successful [108].

## 5. Conclusions

Esophageal varices are a common public health issue with variable prevalence worldwide and account for one of the most frequent causes of death from UGIB. Currently, data from the literature provide data on the most effective treatments for the management of EV.

Primary prophylaxis still has a key role in the prevention of acute bleeding and must be applied by clinical providers. However, this depends on proper clinical evaluation, a correct diagnosis of ACLD, the accurate staging of hepatic fibrosis, and regular follow-ups.

In the case of acute bleeding, a combined medical and endoscopic approach is essential. In this regard, since medical and endoscopic therapy are sometimes managed by different physicians, coordinated patient care is crucial.

Furthermore, it is essential that the endoscopist undergoing the procedure has sufficient experience in the management of esophagogastric varices to minimize adverse outcomes. Moreover, if the medical center does not routinely perform this procedure, patients should be referred to a tertiary care center with endoscopic and IR expertise.

Finally, the management of the patient after the acute bleeding episode and the follow-up in the following weeks is very crucial for preventing re-bleeding, initiating secondary prophylaxis, and ensuring the complete eradication of EV using endoscopy.

More research will be needed in the future to determine the role of hemostatic powder in the treatment of acute and refractory variceal bleeding, as well as the cost-effectiveness of SEMS. Moreover, the role of pre-emptive TIPS in patients with gastric varices needs to be better explored. Additionally, the management of patients with re-compensated cirrhosis needs to be assessed with concomitant guidelines.

To this end, it should be important to estimate the regression of varices after the primary etiological factor is removed or suppressed. Finally, the association between a low platelet count or fibrinogen and the risk of variceal bleeding, failure to control bleeding, or bleeding after endoscopic band ligation must be studied.

## Figures and Tables

**Table 1:** Causes of noncirrhotic portal hypertension.

PREHEPATIC	INTRAHEPATIC	POSTSINUSOIDAL
- Portal vein thrombosis - Splenic vein thrombosis - Splenomegaly due to other causes (e.g., Gaucher’s disease, lymphoma)	**Presinusoidal** - Schistosomiasis - Idiopathic portal hypertension - Primary biliary cholangitis - Sarcoidosis - Congenital hepatic fibrosis - Primary sclerosing cholangitis - Hepatic arteriopetal fistula - Adult polycystic liver disease - Arteriovenous fistulas - Autoimmune cholangiopathy - Peliosis hepatis - Neoplastic occlusion of the intrahepatic portal vein	- Inferior vena cava obstruction (e.g., Budd–Chiari syndrome)
	**Sinusoidal** - Arsenic poisoning - Vinyl chloride toxicity - Drugs (e.g., amiodarone, methotrexate) - Alcoholic liver disease - Nonalcoholic fatty liver disease - Acute fatty liver of pregnancy - Acute hepatic injury - Gaucher’s disease - Viral hepatitis - Schistosomiasis - Amyloidosis - Mastocytosis - Agnogenic myeloid metaplasia - Chronic Q fever	
	**Postsinusoidal** - Budd–Chiari syndrome - Sinusoidal obstruction syndrome (veno-occlusive disease) - Alcoholic liver disease - Chronic radiation injury - Angiosarcoma - Hemangioendothelioma - Sarcoidosis	

**Table 2:** Classification of esophageal varices according to the Japanese Research Society for Portal Hypertension (JRSPH) [12].

Form	**F1**: straight-shaped varices (do not disappear with insufflation) **F2**: slightly enlarged tortuous varices occupying less than one-third of the esophageal lumen **F3**: large-sided varices occupying more than one-third of the esophageal lumen
Fundamental color	**White** (CW) **Blue** (CB)
Red color sign (RC)	**Red Wale Marking** (RWM) **Cherry Red Spot** (CRS) **Hematocystic Spot** (HS) **Diffuse Redness** (DR)
Location	**Locus superior** (Ls): varices located above the level of the tracheal bifurcation **Locus medialis** (Lm): varices located at or near the level of the tracheal bifurcation **Locus inferiorior** (Li): varices located within the area encompassing the abdominal and lower thoracic esophagus
Esophagitis	**Esophagitis positive** (E+) **Esophagitis negative** (E−)

**Table 3:** Classification of gastric varices according to Sarin Classification [13].

**Gastroesophageal varices (GOV)**	**GOV 1:** gastroesophageal varices extended below the gastroesophageal junction along the lesser curvature of the stomach (as a continuation of esophageal varices that are always present) **GOV 2:** gastroesophageal varices extended below the gastroesophageal junction into the fundus of the stomach (as a continuation of esophageal varices that are always present)
**Isolated gastric varices** **(IGV)**	**IGV 1:** isolated gastric varices located in the fundus of the stomach and fall off the cardia by a few centimeters **IGV 2:** isolated ectopic varices appearing in other locations of the stomach (body, antrum, pylorus) or in the duodenum.

**Table 4:** Screening and surveillance intervals for endoscopy in patients with ACLD.

Esophageal Varices	Liver Injury Status	Endoscopy Interval
Absent	Quiescent/absence of risk factors	3 years
	Ongoing	2 years
Small	Quiescent/absence of risk factors	2 years
	Ongoing	1 year

## Data Availability

Not applicable.

## Abbreviations

Acute variceal bleeding (AVB); esophageal varices (EV); upper gastrointestinal bleeding (UGIB); clinically significant portal hypertension (CSPH); portal hypertension (PH); computed tomography (CT); magnetic resonance imaging (MRI); advanced chronic liver disease (ACLD); hepatic venous pressure gradient (HVPG); hepatic vein (HV); free hepatic vein pressure (FHVP); wedged hepatic vein pressure (WHVP); gastric varices (GV); esophagogastroduodenoscopy (EGD); gastroesophageal varices (GOV); isolated gastric varices (IGV); nonselective beta-blockers (NSBB); artificial intelligence (AI); convolutional neural network (CNN); video capsule endoscopy (VCE); endoscopic ultrasound (EUS); fine-needle aspiration (FNA); fine-needle biopsies (FNB); spleen stiffness measurement (SSM); porto-sinusoidal vascular disorder (PSVD); endoscopic band ligation (EBL); cyanoacrylate (CYA); balloon-occluded retrograde transvenous obliteration (BRTO); balloon-occluded antegrade transvenous obliteration (BATO); transjugular intrahepatic portosystemic shunt (TIPS); non-anesthesiology sedation (NAS); Monitored Anesthesia Care (MAC); Non-Operating Room Anesthesia (NORA); Chronic Liver Failure–Sequential Organ Failure Assessment (CLIF-SOFA); acute-on-chronic liver failure (ACLF); area under the receiver operating characteristic curve (AUROC); randomized controlled trials (RCTs); interventional radiology (IR); esophageal variceal sclerotherapy (EVS); endoscopic tissue adhesives (ETA); hemostatic powders (HP); balloon tamponade (BT); self-expandable metal stents (SEMS); polytetrafluoroethylene (PTFE).
